# Supplementary material for: Salt-Induced Changes in Cytosolic pH and Photosynthesis in Tobacco and Potato Leaves
Source: Int J Mol Sci. 2022 Dec 28;24(1):491. doi: 10.3390/ijms24010491 (PMC9820604; doi:10.3390/ijms24010491)
Supplement: Supplementary file 1 [file ijms-24-00491-s001.zip › Figure s2.pdf]

International Journal of
Molecular Sciences

MDPI

Supplementary material

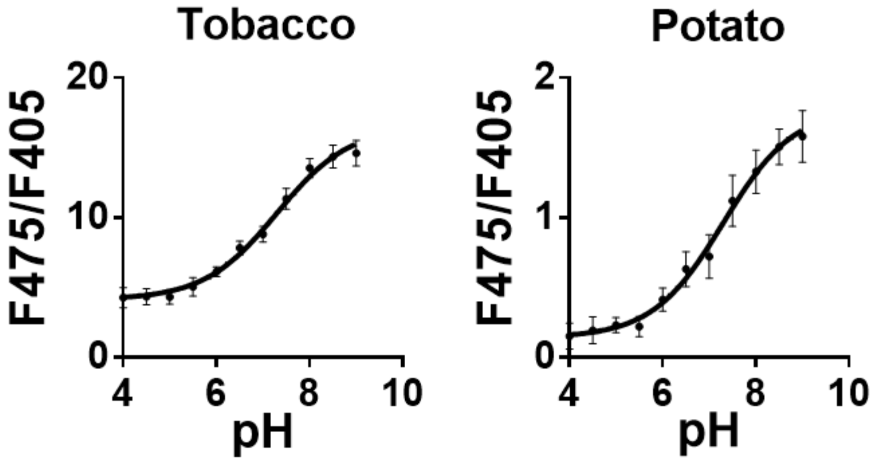

Figure S2. Calibration dependence of Pt-GFP fluorescence of leaf cytosolic pH. Data are represented as mean ± SEM (n = 18).
