# Supplementary material for: Salt-Induced Changes in Cytosolic pH and Photosynthesis in Tobacco and Potato Leaves
Source: Int J Mol Sci. 2022 Dec 28;24(1):491. doi: 10.3390/ijms24010491 (PMC9820604; doi:10.3390/ijms24010491)
Supplement: Supplementary file 1 [file ijms-24-00491-s001.zip › Figure s3.pdf]

International Journal of
Molecular Sciences
MDPI

Supplementary material

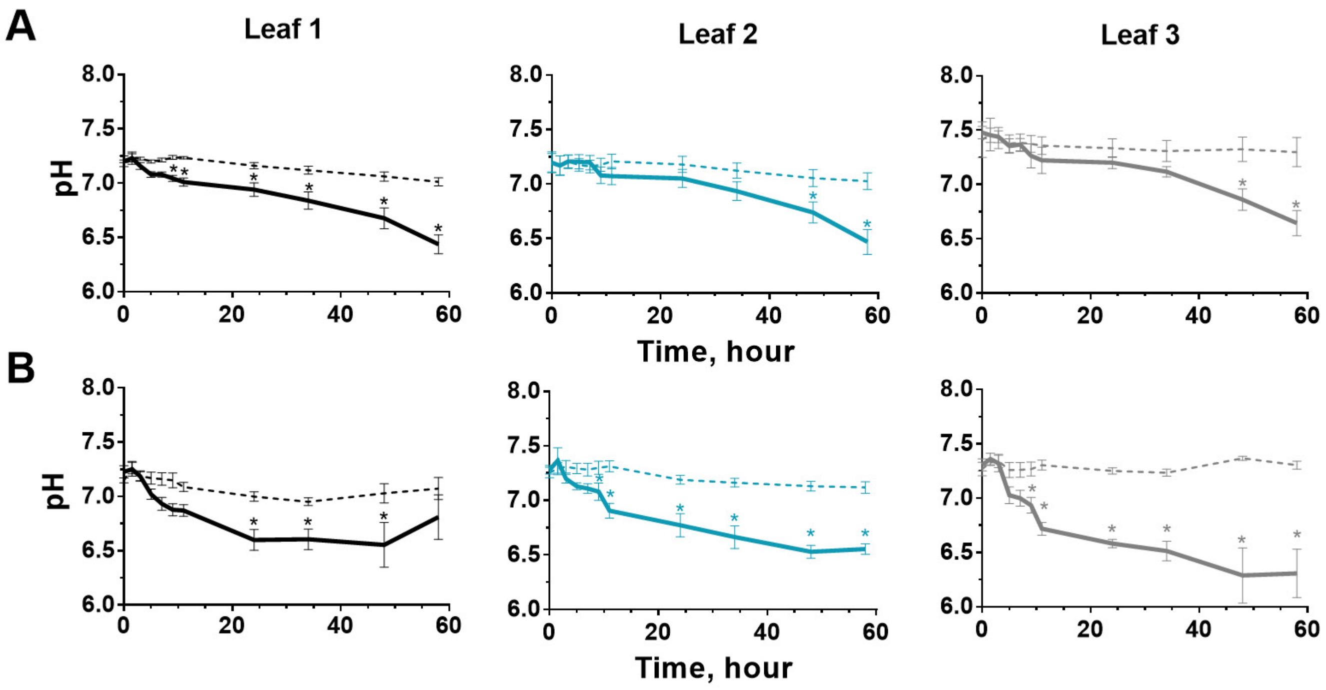

Figure S3. Change of cytosolic pH in leaves of different stratum in tobacco (A) and potato (B) treated by salt (solid) or water (doted). Data are represented as mean ± SEM (n = 9), *p<0.05 control versus salt treatment.
