# Supplementary material for: Salt-Induced Changes in Cytosolic pH and Photosynthesis in Tobacco and Potato Leaves
Source: Int J Mol Sci. 2022 Dec 28;24(1):491. doi: 10.3390/ijms24010491 (PMC9820604; doi:10.3390/ijms24010491)
Supplement: Supplementary file 1 [file ijms-24-00491-s001.zip › Figure s4.pdf]

International Journal of
Molecular Sciences

MDPI

Supplementary material

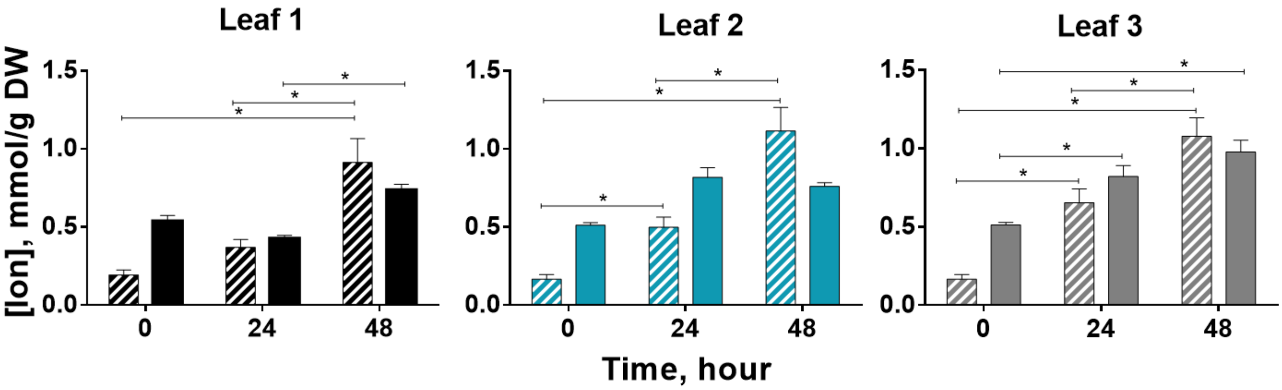

Figure S4. Dynamics of sodium (nonsolid) and chloride (solid) accumulation in tobacco leaves of different stratum (1, 2, 3) during salt treatment. Data are represented as mean ± SEM (n = 6), *p<0.05 between the two parameters.
