# Supplementary material for: Salt-Induced Changes in Cytosolic pH and Photosynthesis in Tobacco and Potato Leaves
Source: Int J Mol Sci. 2022 Dec 28;24(1):491. doi: 10.3390/ijms24010491 (PMC9820604; doi:10.3390/ijms24010491)
Supplement: Supplementary file 1 [file ijms-24-00491-s001.zip › Figure s6.pdf]

International Journal of
Molecular Sciences

Supplementary material

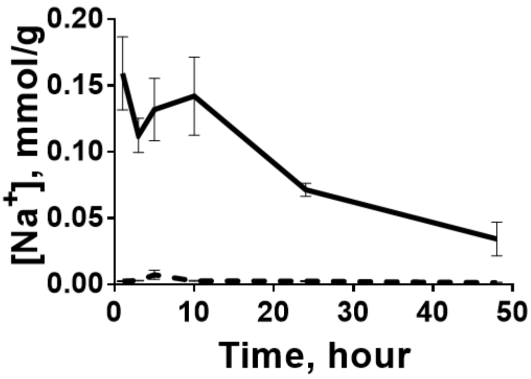

Figure S6. Dynamics of sodium content in soil during salt treatment (salinity – solid, control – dotted). Data are represented as mean ± SEM (n = 6).
