# Supplementary material for: Salt-Induced Changes in Cytosolic pH and Photosynthesis in Tobacco and Potato Leaves
Source: Int J Mol Sci. 2022 Dec 28;24(1):491. doi: 10.3390/ijms24010491 (PMC9820604; doi:10.3390/ijms24010491)
Supplement: Supplementary file 1 [file ijms-24-00491-s001.zip › Table s1.pdf]

International Journal of
Molecular Sciences

MDPI

Supplementary material

Table S1. Changes in photosynthesis parameters in tobacco leaves of different stratum (1, 2, 3) in control (water-treated) and salinity conditions.

Parameter	Leaf	0 h		24 h		48 h	
		Control	Salinity	Control	Salinity	Control	Salinity
F_v/F_m	1	0.785±0.004	0.790±0.003	0.786±0.004	0.774±0.003	0.782±0.004	0.765±0.003*
	2	0.799±0.002	0.795±0.003	0.801±0.002	0.783±0.004*	0.798±0.003	0.774±0.005*
	3	0.802±0.002	0.803±0.002	0.804±0.002	0.791±0.003*	0.803±0.002	0.784±0.004*
Φ_{PSII}	1	0.453±0.012	0.463±0.010	0.397±0.016	0.316±0.016*	0.377±0.013	0.274±0.012*
	2	0.565±0.013	0.546±0.014	0.515±0.016	0.398±0.024*	0.493±0.019	0.339±0.017*
	3	0.602±0.013	0.590±0.012	0.549±0.015	0.430±0.023*	0.540±0.017	0.371±0.019*
NPQ	1	0.702±0.047	0.742±0.044	0.817±0.055	1.069±0.035*	0.824±0.049	1.069±0.015*
	2	0.493±0.041	0.450±0.036	0.510±0.043	0.835±0.058*	0.549±0.051	0.869±0.029*
	3	0.311±0.023	0.315±0.028	0.368±0.025	0.681±0.079*	0.369±0.033	0.771±0.072*
CHL-Ind	1	0.637±0.020	0.694±0.028	0.612±0.027	0.656±0.032	0.591±0.020	0.628±0.031
	2	0.692±0.015	0.747±0.033	0.680±0.023	0.705±0.035	0.665±0.029	0.680±0.030
	3	0.757±0.019	0.757±0.036	0.703±0.033	0.740±0.040	0.671±0.044	0.704±0.036

Data are represented as mean ± SEM (n = 9), *$p<0.05$ control versus salt treatment.
