# Supplementary material for: Salt-Induced Changes in Cytosolic pH and Photosynthesis in Tobacco and Potato Leaves
Source: Int J Mol Sci. 2022 Dec 28;24(1):491. doi: 10.3390/ijms24010491 (PMC9820604; doi:10.3390/ijms24010491)
Supplement: Supplementary file 1 [file ijms-24-00491-s001.zip › Table s2.pdf]

International Journal of *Molecular Sciences*

MDPI

Supplementary material

Table S1. Changes in photosynthesis parameters in potato leaves of different stratum (1, 2, 3) in control (water-treated) and salinity conditions.

Parameter	Leaf	0 h		24 h		48 h	
		**Control**	**Salinity**	**Control**	**Salinity**	**Control**	**Salinity**
F_v/F_m	1	0.796±0.005	0.796±0.003	0.781±0.007	0.729±0.008	0.745±0.015	0.695±0.017
	2	0.789±0.009	0.806±0.004	0.774±0.008	0.753±0.010	0.731±0.027	0.688±0.018
	3	0.802±0.004	0.811±0.002	0.779±0.009	0.771±0.006	0.776±0.005	0.685±0.019*
Φ_{PSII}	1	0.562±0.027	0.588±0.023	0.473±0.032	0.381±0.012	0.394±0.038	0.312±0.026
	2	0.626±0.010	0.611±0.018	0.530±0.025	0.406±0.027*	0.489±0.022	0.273±0.020*
	3	0.603±0.016	0.625±0.013	0.529±0.026	0.399±0.024*	0.532±0.018	0.290±0.026*
NPQ	1	0.374±0.029	0.336±0.042	0.415±0.046	0.569±0.049	0.405±0.045	0.518±0.073
	2	0.268±0.042	0.308±0.033	0.368±0.048	0.589±0.052*	0.350±0.031	0.670±0.069*
	3	0.243±0.036	0.285±0.027	0.282±0.032	0.631±0.042*	0.300±0.043	0.535±0.052*
CHL-Ind	1	0.816±0.056	0.756±0.018	0.756±0.056	0.630±0.037	0.664±0.062	0.557±0.029
	2	0.704±0.043	0.760±0.017	0.711±0.040	0.707±0.022	0.649±0.049	0.549±0.034
	3	0.673±0.044	0.732±0.05	0.661±0.025	0.699±0.029	0.677±0.022	0.570±0.041

Data are represented as mean ± SEM (n = 9), $*p<0.05$ control versus salt treatment.
